# Regulation of body temperature and blood pressure in women: Mechanisms and implications for heat illness risk

**DOI:** 10.1113/EP091455

**Published:** 2024-04-12

**Authors:** Gabrielle E. W. Giersch, Nisha Charkoudian

**Affiliations:** ^1^ US Army Research Institute of Environmental Medicine Natick Massachusetts USA

**Keywords:** exercise, exertional heat stroke, integrative physiology, sex differences, thermoregulation

## Abstract

Increasing global temperatures due to ongoing climate change phenomena have resulted in increased risk of exertional heat illness in otherwise healthy, young individuals who work or play in the heat. With increasing participation of women in athletic, military and industrial activities that involve exertion in the heat, there is a growing need to study female physiology in this context. Mechanisms controlling blood pressure and body temperature have substantial overlap in humans, largely due to autonomic mechanisms which contribute to both. Similarly, illnesses that result from excessive heat exposure can often be traced back to imbalances in one or more of these autonomic mechanisms. In recent years, there has been increased recognition of the importance of sex as a biological variable for basic and applied research in these areas. The goal of this paper is to present an update on the integrative physiology and pathophysiology of responses to heat stress in women (thermoregulation and blood pressure regulation). In this context, it is often the case that differences between sexes are presented as ‘advantages’ and ‘disadvantages’ of one sex over the other. In our opinion, this is an over‐simplification of the physiology which ignores the nuances and complexities of the integrative physiology of responses to heat exposure and exercise, and their relevance for practical outcomes.

## Abstract (highlights)

- **What is the topic of this review?**
  Integrative mechanisms of body temperature and blood pressure regulation in women.
- **What advances does it highlight?**
  A framework is provided for conceptualizing the integrative nature of blood pressure regulation and thermoregulation in women as dynamic and circumstance‐specific. In almost all situations, any ‘advantage’ for one sex could also be a ‘disadvantage’ if conditions were altered.

## 1 INTRODUCTION

Because of the ongoing (and escalating) threat of climate change, global temperatures continue to increase, and the number and severity of heat waves continue to rise (Molina et al., 2020). For people who participate in regular outdoor activities, including for athletic, military or industrial purposes, one consequence is a progressive augmentation in risk of serious heat illness. Heat illness exists on a spectrum, from relatively mild heat exhaustion to the most serious, exertional heat stroke (EHS). EHS is a life‐threatening emergency that requires prompt cooling intervention, immediate hospitalization and can lead to long term organ damage and sometimes death ((Roberts et al., 2021). Women represent a growing proportion of the people who participate in activities that put them at risk but are markedly under‐represented in the research in this area (Cowley et al., 2021; James et al., 2023)

## 2 AUTONOMIC CONTROL OF BLOOD PRESSURE AND BODY TEMPERATURE

The autonomic nervous system is ubiquitous in its control of blood flow via sympathetic innervation of the vasculature, and thus is a key contributor to regulation of body temperature and arterial blood pressure via this mechanism. The autonomic nervous system controls arterial pressure primarily via the arterial baroreflex, a negative feedback system which relies on afferent information from aortic, carotid and cardiopulmonary mechanoreceptors to send information to central autonomic nuclei regarding the prevailing arterial pressure. This information is integrated and elicits reflex changes in efferent sympathetic and parasympathetic activity which control heart rate, stroke volume and total peripheral resistance. For more comprehensive discussion of these integrative mechanisms, the interested reader is referred to several extensive reviews of the autonomic regulation of mean arterial blood pressure (Hart & Charkoudian, 2014; Wehrwein & Joyner, 2013).

Sympathetic and parasympathetic innervation of the heart also contribute to the changes in cardiac output required to appropriately respond to activity in a hot environment. Both the physiology and pathophysiology of thermoregulation in the heat are complex, integrative phenomena, in which the cardiovascular system has a central, coordinating role. Blood flow to various organs and tissues must balance thermoregulatory requirements with that for metabolic and blood pressure regulatory processes. Normal thermoregulation in the heat requires increases in cardiac output (CO; proportional to the severity of heat stress and thermoregulatory needs) to support the increase in skin blood flow that is required for convective transfer of heat from the core to the periphery. Increased body temperature also activates sympathetic sudomotor (cholinergic) nerves, which increase sweating rate (Periard et al., 2016). The evaporation of sweat cools the skin, which then works in concert with the increased skin blood flow to dissipate heat to the environment. Interestingly, sympathetic vasoconstrictor (noradrenergic) nerves increase in activity in response to heat stress and help to maintain blood pressure despite the lower relative venous return (Schlader et al., 2016). Additionally, environments with high humidity represent increased risk for excessive hyperthermia since it is more difficult for sweat to evaporate when the vapor pressure of water in the air is already elevated. The requirements for cardiac output are further stretched during exercise scenarios, when working muscle has increased blood and oxygen requirements and balance must be struck between the skin for heat dissipation and the working muscles for maintenance of movement (Johnson et al., 1973).

## 3 SEX AS A BIOLOGICAL VARIABLE IN THERMOREGULATION AND BLOOD PRESSURE REGULATION

**FIGURE 1:**
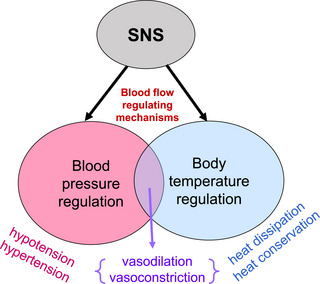
Schematic representation of the overlap between blood pressure regulatory mechanisms and thermoregulatory mechanisms, both of which involve sympathetic neural control of blood flow. This is particularly true during heat stress, where vasodilation promotes heat dissipation and lower blood pressure, and vasoconstriction promotes higher body temperature and higher blood pressure.

In young women, there is an oestradiol‐mediated augmentation of peripheral blood flow, which likely contributes to lower blood pressures (on average) compared to young men (Joyner et al., 2016). Furthermore, young women tend to have lower blood pressure and blunted neurovascular transduction of sympathetic nerve activity into vasoconstriction (Charkoudian et al., 2017). Interestingly, the mechanisms which may help promote lower blood pressure in younger, pre‐menopausal women (e.g., oestradiol‐mediated augmentation of peripheral vasodilation) may also modulate thermoregulatory responses (Figure 1) (Charkoudian et al., 2017). Aerobic fitness may also influence these relationships, since higher fitness levels improve thermoregulatory responses (Mora‐Rodriguez, 2012), and can also blunt autonomic support of arterial pressure in young women (Baker et al., 2020).

When people think of sex as a biological variable in physiological responses, they often think in terms of sex differences and potential ‘advantages’ or ‘disadvantages’ that men or women might have. In terms of the integrative physiology of thermoregulation and blood pressure regulation, this type of categorization is often an over‐simplification of processes that are very situation‐specific and involve significant inter‐individual variability in multiple factors simultaneously. For example, it is sometimes stated that young women (before menopause) have an ‘advantage’ in terms of blood pressure regulation compared to men of similar age (Joyner et al., 2016). It is true that population averages often show women have lower blood pressure—however, some women have high blood pressure, just as some men have low blood pressure. Furthermore, a tendency towards lower blood pressure is not always an advantage, since women are 5:1 more likely to develop so‐called ‘hypotensive’ disorders like postural tachycardia syndrome and orthostatic hypotension (Ali et al., 2000).

## 4 ‘ADVANTAGES’ OR ‘DISADVANTAGES’ ARE SPECIFIC TO THE CIRCUMSTANCE

The idea of ‘advantages’ and ‘disadvantages’ is also pervasive in the literature regarding thermoregulation. Sometimes researchers are asked by leadership in military or athletic scenarios, who is better/who has the advantage? These are reasonable questions, but as with blood pressure regulation, the answer(s) are more nuanced. For example, women, who are on average smaller, have been proposed to be at an ‘advantage’ for heat dissipation due to inherent biophysical differences of smaller body size (Shapiro et al., 1980; Taylor et al., 2024). However, when performing high intensity exercise, load carriage, or in environmental or clothing conditions that impede heat dissipation, being smaller and/or having a higher body surface area:mass ratio can lead to increased heat *gain* from the environment or other factors—and would therefore become a ‘disadvantage’ in a dichotomous model.

Similarly, women have often been suggested to sweat less, and while there is evidence to suggest lower sweat output per gland and local sweat rate, this does not necessarily suggest that women are not able to sweat adequately for the given evaporative requirement, rather that women are sweating less at a similar requirement for heat loss (Gagnon & Kenny, 2012; Gagnon et al., 2013). While core temperatures were slightly elevated in the women in these investigations, this small physiological difference likely would not contribute to a practical difference in outcome, particularly as it relates to heat illness. Additionally, in certain environments where sweat evaporation is hindered due to high humidity, and sweat is simply dripping off the skin, the increased sweat rate results in excessive fluid loss and potential dehydration, without adding a thermoregulatory benefit at all. Previously, it was observed that women have a lower sweat rate in high humidity conditions thought to be caused by an increased sensitivity to hidromeiosis (local inhibition of sweating due to high sweating rates) (Shapiro et al., 1980). This has also been previously postulated to be an ‘advantage’ for women, where in other circumstances, sweating less regardless of environmental conditions would be viewed as detrimental to heat dissipation and overall thermoregulation. These various factors and circumstances appear to balance each other out; we recently showed that overall EHS risk was similar between men and women in a group of ∼4500 healthy soldiers over a 10‐year period (Giersch et al., 2023).

There is also a breadth of interesting work in the murine model on the influences of female sex hormones on exertional heat stroke risk (Rentería et al., 2023). Previously, it has been observed that female mice were able to withstand longer running times and higher temperatures than male mice (Garcia et al., 2018). In a subsequent study, Rentería et al. (2023) performed ovariectomy on female mice and compared them to non‐ovariectomized mice, observing a similar difference in response of male and female mice to ovariectomized and non‐ovariectomized mice. This suggests that the female sex hormones are somehow protective in these mice. How this translates to a human model is currently unclear, and more research is in progress and needed to fully elucidate the influence or any possible benefit of female sex hormones on EHS risk in women, as well as any possible overlap in mechanisms related to female sex hormones, as previously discussed. Recently, Kirby et al. (2024) reported that progestin‐only intrauterine devices did not alter dry or evaporative heat loss during light or moderate intensity exercise in the heat, providing helpful new information in this ongoing discussion. This and other future work will be valuable for women who are utilizing hormonal contraception (exogenous supplementation), have amenorrhoea or are engaging in menstrual suppression (menstrual dysfunction), or who are in perimenopause or menopause.

## 5 SUMMARY AND CONCLUSIONS

Although there is a relative dearth of research into the integrative physiological responses to exertional heat stress in women, available data point to distinct differences between men and women. These differences can include blood pressure, blood flow, sweating and body size/biophysical aspects. Our main emphasis here is on the fact that these must be taken in context of the specifics of the circumstance in question, to determine whether any such differences are helpful or not. Overall, in terms of group comparisons of men versus women, it appears that any such mechanistic differences do not result in group ‘net’ differences in heat illness risk between men and women.

## AUTHOR CONTRIBUTIONS

All authors have approved the final version of the manuscript and agree to be accountable for all aspects of the work in ensuring that questions related to the accuracy or integrity of any part of the work are appropriately investigated and resolved. All persons designated as authors qualify for authorship, and all those who qualify for authorship are listed.

## CONFLICT OF INTEREST

The views, opinions, and/or findings contained in this article are those of the authors and should not be construed as an official United States Department of the Army position, or decision, unless so designated by other official documentation. Approved for public release, distribution unlimited. Citations of commercial organizations and trade names in this report do not constitute an official Department of the Army endorsement or approval of the products or services of these organizations.

## FUNDING INFORMATION

No funding was received for this work.
